# Systemic and tumor-specific inflammatory markers VCAM-1 and ICAM-1 as indicators of extent of surgery and oncologic outcome in advanced ovarian cancer

**DOI:** 10.1016/j.tranon.2025.102462

**Published:** 2025-07-12

**Authors:** Okan Gultekin, Jordi Gonzalez-Molina, Dhifaf Sarhan, Nina Groes-Kofoed, Mahmood Ul Hassan, Kaisa Lehti, Sahar Salehi

**Affiliations:** a Department of Women’s and Children’s Health, division of Obstetrics and Gynecology, Karolinska Institutet, Stockholm, Sweden; b Department of Microbiology, Tumor and Cell Biology, Karolinska Institutet, Stockholm, Sweden; c Department of Laboratory Medicine, Division of Pathology, Karolinska Institutet, Stockholm, Sweden; d Department of Biomedical Laboratory Science, Norwegian University of Science and Technology/NTNU, Trondheim, Norway; e Department of Pelvic Cancer, Theme Cancer, Karolinska University Hospital, Stockholm, Sweden; f Division of Biostatistics, Institute of Environmental Medicine, Karolinska Institutet, Stockholm, Sweden

**Keywords:** Advanced ovarian cancer, Cytoreductive surgery, Surgical complexity, Biomarker

## Abstract

**Background:**

Cytoreductive surgery in advanced ovarian cancer presents significant challenges and there is a need for an improved patient selection to surgical treatment. Cytokines and adhesion molecules are key regulators of immune responses, playing crucial roles in tumor cell adhesion, metastasis, and immune evasion. They shape the tumor microenvironment and influence systemic immunity, ultimately affecting cancer progression. Despite their recognized significance in cancer biology, the potential of cytokines as predictive biomarkers for surgical complexity and recurrence in ovarian cancer remains insufficiently characterized. This study aimed to elucidate the correlation between inflammatory cytokines and surgical outcomes, to identify reliable liquid or tissue-based biomarkers that could enhance patient stratification and support preoperative decision-making in ovarian cancer management.

**Methods:**

The Concentration of 10 inflammatory cytokines and 2 adhesion molecules concentrations were measured by Luminex based assay in blood, tumor tissue, and ascitic fluid samples from patients with advanced ovarian cancer prior to cytoreductive surgery. Clinical data were prospectively collected. Correlations between cytokines and adhesion molecules levels and surgical complexity, as well as disease/cancer recurrence, were assessed using Pearson two-tail statistical test analyses. The association between adhesion molecules and surgical extent, and recurrence was analysed using logistic regression yielding odds ratios (OR) with 95 % confidence intervals, adjusted for relevant covariates. The diagnostic accuracy of biomarker candidates was evaluated using receiver operating characteristic (ROC) curve analysis

**Results:**

In blood, higher VCAM-1 and ICAM-1 levels correlated with lower surgical complexity scores, while ascitic VCAM-1 was linked to longer surgical durations. CXCL-12 in tumor and IL-32 in ascites were positively correlated with increased surgery duration, indicating their role in systemic inflammation. Elevated VCAM-1 and ICAM-1 levels in tumor tissue were strongly associated with increased cancer recurrence risk, suggesting their involvement in metastasis and immune evasion. Traditional preoperative markers, including albumin and CRP, did not correlate significantly with surgical complexity, highlighting the need for novel biomarkers. In the adjusted multivariable regression model, VCAM-1 in blood was associated with recurrence, OR 10.1 (95 % CI, 1.30-77.8; *p*=0.027). Similarly, VCAM-1 in blood demonstrated exceptional predictive capability, Area Under Curve=0.886 with cutoff point of 0.696.

**Conclusions:**

Inflammatory markers can serve as valuable predictors of surgical complexity and recurrence in advanced ovarian cancer. Particularly the levels of VCAM-1 in blood was identified as a potential predictive marker to be tested in adequately powered clinical studies. Incorporating these markers into preoperative assessments could improve surgical planning and enhance patient stratification. Further validation and mechanistic studies are needed to fully understand their role in ovarian cancer progression.

**Trial registration:**

ClinicalTrials.gov nr: NCT04065009, European Union Clinical Trials Register nr: 2019-003299-38/SE

## Highlights

• Cytokines and adhesion molecules may have potential as biomarkers predicting clinical outcomes in advanced ovarian cancer.
• The level of VCAM-1 in blood demonstrated exceptional capability of predicting disease recurrence.
• If our results are validated in adequately powered prospective studies, integrating these identified biomarkers into preoperative evaluation could refine patient selection to cytoreductive surgery in advanced ovarian cancer.

## Background

Most patients with epithelial ovarian cancer are diagnosed at an advanced stage with tumor spread throughout the peritoneal cavity and presenting extensive peritoneal carcinomatosis [1]. Notwithstanding multimodal treatments including cytoreductive surgery, chemotherapy, and targeted therapies, the prognosis remains poor [[2], [3], [4]]. Surgical cytoreduction plays a pivotal role in improving survival by enhancing the impact of adjuvant chemotherapy [[5], [6], [7], [8]]. Repeated observations have consistently suggested a significant association between the amount of residual tumor following cytoreductive surgery and patient survival, with a smaller amount—or preferably no residual tumor—being associated with a more favorable prognosis [9,10]. This clinical association, combined with the theoretical basis behind the effect of cytoreductive surgery, forms the foundation that motivates extensive surgical efforts to achieve complete macroscopic resection [[11], [12], [13], [14], [15]]. However, a larger preoperative tumor burden, which necessitates more extensive surgical intervention to achieve complete macroscopic resection, is also associated with a poorer prognosis. The impact of initial tumor burden and the surgical complexity needed for complete macroscopic resection on survival outcomes has been largely underestimated.

We previously showed that there is a limit to when extensive surgery cannot outweigh the disadvantage of a more extensive tumour dissemination, despite achieving complete macroscopic resection [16]. Accordingly, there is an urgent need to improve patient selection to surgical treatment, by identifying women in whom a high surgical complexity/extent is needed to achieve complete macroscopic resection *before* the surgical procedure. However, this task presents a significant challenge as there is no imaging modality that may accurately predict the extent of peritoneal carcinomatosis, and extent of surgery needed to achieve complete macroscopic resection.

To address these challenges, the identification of a reliable liquid or tissue-based biomarker that can be evaluated prior to treatment decisions is crucial. Such biomarkers would provide essential insights into the surgical extent required to achieve optimal outcomes, thereby enhancing patient selection and preoperative planning [17,18].

Cytokines and adhesion molecules orchestrate immune regulation, inflammation, and tumor progression. Cytokines, secreted by immune and tumor cells, modulate the tumor microenvironment, influencing metastasis, immune evasion, and therapy resistance. Inflammatory cytokines, consistently elevated in cancer patients, correlate with tumor dissemination and poor prognosis. The adhesion molecules VCAM1 and ICAM1 guide immune cell transmigration, with ICAM1 favoring neutrophils, macrophages, and lymphocytes, while VCAM1 primarily recruits monocytes and lymphocytes, ultimately regulating the tumor immune microenvironment [19,20]. Similarly, various chemokines and cytokines regulate immune cell migration, activation, proliferation as well as tumor invasion, metastasis, and angiogenesis [18,[21], [22], [23]]. All of which are central functions in tumor development and progression highlighting their potential role as biomarkers to predict tumour dissemination. Ultimately with potential to optimize patient stratification to treatment [18,[21], [22], [23]].

Despite the promising implications of cytokines as biomarkers and therapeutic targets, current data on their correlation with clinical outcomes remains limited [[24], [25], [26]]. For this reason, our aim was to comprehensively explore whether inflammatory markers in the blood, tumour biopsies and ascites retrieved prior to the cytoreductive surgical procedure in advanced ovarian cancer could serve as potential markers for tumour dissemination and the extent of surgery needed to achieve complete macroscopic resection.

## Methods

### Patients and sample collection

All samples were collected from women who underwent upfront cytoreductive surgery for advanced epithelial ovarian cancer with curative intent, included in the multicenter national IPLA-OVCA phase III clinical trial conducted during 2020-2023 [27]. The aim and results of this clinical trial have been reported previously [27].

Blood, omental tumor and peritoneal biopsies were collected immediately after incision and entry of the abdomen before the surgical cytoreductive procedure began in 40 participating patients. The samples were immediately collected from the operation theater and processed. All clinical data including surgical complexity was prospectively entered in the IPLA-OVCA study database, monitored according to good clinical practice as stated in Alletti et al [28]. The surgical team was blinded to the results of the biomarker analysis.

All women consented to participation. The study was approved by the Swedish Ethical Review Authority (Dnr: 2019-05149), the Swedish Medical Products Agency (Dnr: 5.1-2019-85294). It was registered at ClinicalTrials.gov (nr: NCT04065009, August 2019) and at the European Union Clinical Trials Register (nr: 2019-003299-38/SE, November 2019).

### Sample preparations

#### Ascites

Fresh ascitic fluid samples were collected and divided into two 1.5 mL RNase/DNase-free Eppendorf tubes. Samples were centrifuged at 800 x g for 10 minutes, and the supernatant was carefully transferred to two additional 1.5 mL RNase/DNase-free Eppendorf tubes. All four tubes were immediately frozen on dry ice and subsequently stored at -80°C for preservation.

#### Blood

Blood samples were mixed thoroughly by inverting the collection tubes 8–10 times. Samples were then centrifuged at 2000 × g for 15 minutes at room temperature (RT), with both acceleration and braking set to “Slow” to prevent disruption of blood fractions. Following centrifugation, the plasma fraction (top layer) was carefully transferred to 1.5 mL DNA LoBind tubes, with approximately 1.2–1.3 mL of plasma per tube, yielding 20 tubes per sample (excluding tube #1). Care was taken to avoid disturbing the buffy coat (cloudy, thin middle layer) during plasma transfer.

The plasma fractions were subsequently centrifuged at 10,000 × g for 10 minutes at RT. Supernatants were then transferred to new 1.5 mL DNA LoBind tubes. All samples were stored at -80°C for future analyses.

#### Tumor

Tumor tissue samples (approximately 20–50 mg) were rinsed briefly in cold phosphate-buffered saline (PBS) to remove blood and contaminants, then gently patted dry with sterile, RNase/DNase-free paper towels. Each tissue was placed in a pre-chilled 1.5 mL microcentrifuge tube with 300–500 µL of cold protein extraction buffer (RIPA buffer with protease and phosphatase inhibitors) and homogenized thoroughly using a tissue homogenizer. Homogenized samples were vortexed for 15–30 seconds and incubated on ice for 30 minutes, with vortexing every 10 minutes to ensure complete lysis. Lysates were centrifuged at 14,000 × g for 20 minutes at 4°C, and the supernatant was carefully transferred to a fresh, pre-chilled microcentrifuge tube, taking care to avoid the pellet. For higher purity, a second centrifugation of the supernatant at 14,000 × g for 10 minutes at 4°C was performed. Protein concentrations were determined using a BCA Protein Assay Kit, following the manufacturer’s instructions. Protein extracts were aliquoted and stored at -80°C to avoid repeated freeze-thaw cycles and remained stable for several months under these conditions.

### Measurement of serum, tumor, and ascites cytokine levels

The cytokine profiles of serum, tumor and ascites were determined using Bio-Rad fluorescently labeled magnetic microspheres and the Bio-Plex 200 Systems instrument. The screening was performed for a total of 10 cytokines and 2 adhesion molecules in the following batches: VEGF, CCL-2/MCP-1, CXCL-12/SDF-1⍺, IL-6, CXCL-1/GRO⍺, CCL-5/RANTES, CXCL-8/IL-8, IL-16 were analyzed according to Bio-Plex Pro Human Cytokine Screening Panel manual (kit #10000092045), SerpinE1/PAI-1 according to Bio-Plex Pro Human Diabetes Assays manual (kit #10000094511), IL-32 according to Bio-Plex Inflammation Panel 1 manual (kit #171304090M) and ICAM-1 and VCAM-1 according to Bio-Plex Pro Human Cytokine Screening Panel manual (kit #10000092045). The magnetic beads, detection antibodies and cytokine assay kits were ordered from Bio-Rad. The magnetic bead washing steps were performed via Bio-Plex Pro™ Wash Station, in accordance with manual instructions. The calibration of the Bio-Plex 200 Systems instrument was done prior to every analysis via the Bio-Plex Calibration Kit (#171203060). Validation of the instrument was performed monthly via the Bio-Plex® Validation Kit 4.0 (#171203001).

### Statistical analyses

Descriptive statistics were calculated and visualized using figures. To compare cytokine levels, non-parametric ANOVA (Kruskal–Wallis test) was used for within-group comparisons, while one-way ANOVA was applied for intragroup comparisons. Distributional differences by recurrence and surgical extent was tested using the Wilcoxon rank sum exact test, Wilcoxon rank sum test and Fisher's exact test.

The correlation between cytokine levels and clinical outcomes was assessed using the two-tailed Pearson correlation coefficient. Follow-up time was estimated using the reversed Kaplan–Meier method.

To evaluate the independent associations of cytokine levels with clinical outcomes adjusted for predefined clinical variables, we used multivariate logistic regression. The predefined covariates were factors that may be known prior to the surgical procedure and with relevance for the outcome surgical extent; Preoperative suspected FIGO stage (III *vs.* IV) and for the outcome recurrence: Age (years, continuous), ECOG performance status (0, 1 or 2), BRCA mutation in tumor (yes *vs.* no) Preoperative suspected FIGO stage (III *vs.* IV)

Logistic regression was performed using the mixed bias-reduction adjusted scores approach described by Kosmidis et al. (2020) [29], which provides mean bias reduction for regression coefficients and median bias reduction for the dispersion parameter. This method is particularly suitable for small samples, rare events, and datasets prone to data-separation issues.

The diagnostic accuracy of cytokine levels and logistic regression models (both unadjusted and adjusted) was evaluated using receiver operating characteristic (ROC) curve analysis. Discriminative ability was quantified by area under the curve (AUC), with AUC > 0.8 indicating strong performance. Optimal cutoff thresholds for cytokine levels were determined using Youden’s index to maximize sensitivity and specificity.

All statistical tests were two-sided, with p-values less than 0.05 considered statistically significant. Statistical analyses were performed using Prism v8.0 (GraphPad Software) and R v4.4.1 (R Core Team, 2024).

## Results

### Patient demographics, clinical characteristics, and surgical outcomes

A total of 40 patients with advanced stage high-grade serous ovarian cancer were included in this study, the clinical characteristics are presented in Table 1. The median age was 67 years (IQR: 56–74). 92 % (*n* = 37) had a performance status of 0-1 according to the Eastern Cooperative Oncology Group (ECOG). Most patients had an International Federation of Gynecology and Obstetrics (FIGO) stage IIIC (53 %, *n* = 21), followed by IVB (28 %, *n* = 11), IVA (18 %, *n* = 7), and IIIB (3 %, *n* = 1).

**Table 1:** Clinical-, treatment and tumor characteristics of included patients.

Characteristics	*n* = 40
Age, years Median (IQR)	67 (56-74)
ECOG performance status, no. (%) 0 1 2	22 (55) 15 (38) 3 (8)
Preoperative Serum-Albumin, g/L Median (IQR)	34 (29-36)
Preoperative Plasma-CRP, mg/L Median (IQR)	22 (9-45)
Preoperative Serum-Transthyretin, g/L Median (IQR)	0.16 (0.11-0.24)
Preoperative Plasma-Fibrinogen, g/L Median (IQR)	4.40 (3.80-5.20)
Ascites present at beginning of surgery, no (%) Yes No Volume of ascites at beginning of surgery, mL Median (IQR) Mean (SD)	37 (93) 3 (8) 550 (150-1700) 1208 (1582)
Surgical complexity score^a^ Median (IQR) Surgical complexity score, grouped, no. (%) Low (0-3) Medium ([4], [5], [6], [7]) High (≥8)	9 (6-11) 3 (8) 12 (30) 25 (63)
Operation time, minutes Median (IQR)	336 (276-434)
Intra-abdominal residual disease, no. (%) 0 1-5 6-10 >10	17 (43) 7 (18) 8 (20) 8 (20)
Postoperative histologic subtype, no. (%) High grade serous adenocarcinoma	40 (100)
Definitive FIGO stage, no. (%) IIIB IIIC IVA IVB	1 (3) 21 (53) 7 (18) 11 (28)
Recurrence, no. (%) Yes No	29 (73) 11 (28)

Abbreviations: IQR, Interquartile range; SD, Standard deviation; BMI, Body mass index; ECOG, Eastern Cooperation Oncology group; ASA, American society of Anesthesiologist physical status classification, FIGO, International federation of Gynecology and Obstetrics.

a According to Mayo clinic.

Preoperative clinical serum/plasma inflammatory markers revealed a median serum albumin level of 34 g/L (IQR: 29–36), median C-reactive protein of 22 (IQR: 9-45), median transthyretin of 0.16 g/L (IQR: 0.11–0.24), and a median plasma fibrinogen level of 4.40 g/L (IQR: 3.80–5.20), Table 1. Ascites was present in 93 % (*n* = 37) of patients at the beginning of surgery. Surgical complexity scores were recorded with a median of 9 (IQR: 6–11), and the median operation time was 336 minutes (IQR: 276–434). After a median follow-up time of 34 months (IQR 29-43), recurrence was observed in 73 % (*n* = 29) of patients. (Table 2, Table 3)

**Table 2:** Association between preoperative VCAM-1 and ICAM-1 levels and extent of surgery in patients with advanced ovarian cancer undergoing surgery with curative intent.

	***Unadjusted***		***Adjusted***^1^	
**Variable**	**OR (95% CI)**	**p-value**	**OR (95% CI)**	**p-value**
VCAM-1 in tumour	0.36 (0.05, 2.61)	0.314	0.48 (0.06, 3.74)	0.487
ICAM-1 in tumour	0.08 (0.00, 1.69)	0.104	0.11 (0.00, 2.38)	0.0157
VCAM-1 in blood	0.16 (0.03, 0.96)	**0.045**	0.18 (0.03, 1.06)	0.058
ICAM-1 in blood	0.23 (0.05, 1.11)	0.067	0.23 (0.05, 1.16)	0.075
VCAM-1 in ascites	0.89 (0.29, 2.74)	0.800	0.95 (0.30, 2.99)	0.926

***Abbreviations:*** VCAM-1, Vascular Cell Adhesion Molecule; ICAM-1, Intercellular Adhesion Molecule; OR, Odds Ratio; CI, Confidence Interval; FIGO, International Federation of Gynecology and Obstetrics.

1 Adjusted for the clinical variable that is known prior to surgery and associated with extent of tumour dissemination: preoperative suspected FIGO stage (III *vs.* IV) .

**Table 3:** Association between preoperative VCAM-1 and ICAM-1 levels and recurrence in patient with advanced ovarian cancer undergoing surgery with curative intent.

	***Unadjusted***		***Adjusted***^1^	
**Variable**	**OR (95% CI)**	**p-value**	**OR (95% CI)**	**p-value**
VCAM-1 in tumor	8.74 (0.86-89.3)	0.067	4.74 (0.40-56.7)	0.219
ICAM-1 in tumor	22.1 (0.65-756)	0.086	14.7 (0.25-884)	0.198
VCAM-1 in blood	9.53 (1.80- 50.6)	0.008	10.1 (1.30-77.8)	0.027

***Abbreviations:*** VCAM-1, Vascular Cell Adhesion Molecule; ICAM-1, Intercellular Adhesion Molecule; OR, Odds Ratio; CI, Confidence Interval; ECOG, Eastern Cooperative Oncology Group performance status; BRCA, BReast CAncer gene 1 or 2; FIGO, International Federation of Gynecology and Obstetrics.

1 Adjusted for clinical variables that may be known prior to the surgical procedure: Age (years, continuous), ECOG performance status (0, 1 or 2), BRCA mutation in tumor (yes *vs.* no) Preoperative suspected FIGO stage (III *vs.* IV) .

### Selection of soluble factors to analyze

The cytokines and soluble factors analyzed in this study were selected based on their established roles in tumor progression, immune modulation, and the tumor microenvironment in high-grade serous carcinoma (HGSC). Pro-inflammatory cytokines such as IL-6, IL-8, IL-16, and IL-32 were included due to their involvement in tumor growth, angiogenesis, and chemoresistance. Factors associated with angiogenesis and metastasis, including VEGF and CXCL12, were selected for their contributions to tumor vascularization and dissemination. Immune modulatory molecules, such as CCL2 and CCL5, which recruit tumor-associated macrophages, and adhesion molecules ICAM1 and VCAM1, which facilitate immune cell trafficking, were also included. Additionally, CXCL1 and PAI-1 were analyzed due to their roles in enhancing tumor cell survival, invasion, and resistance to apoptosis. This panel was chosen to provide a comprehensive analysis of key inflammatory, angiogenic, and immune-regulatory pathways relevant to HGSC pathophysiology.

### Distinct inflammatory cytokine and adhesion molecule profiles in tumor, ascites, and blood correlate with surgical complexity and duration in advanced ovarian cancer

**Fig. 1:**
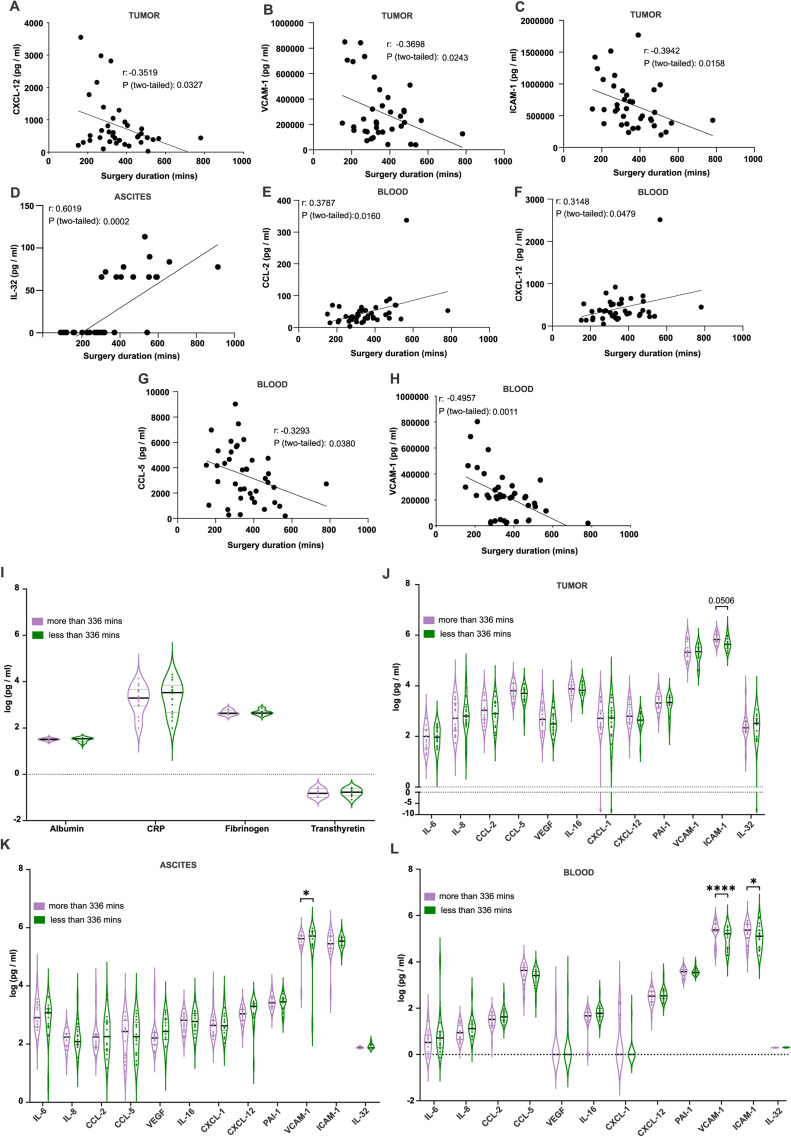
Prolonged surgery duration correlates with specific inflammatory markers in blood, tumor, and ascitic fluid. Bioplex analysis revealed significant differences of VCAM-1, ICAM-1levels in blood (A), only VCAM1 levels in ascites (A-C) Levels of VCAM-1, CXCL12, and ICAM-1 in tumor tissue showed negative correlations with surgery duration. (D) In ascitic fluid, IL-32 exhibited a strong positive correlation with surgery duration. (E-H) Positive correlations were observed between surgery duration and levels of CCL2 and CXCL12 in blood, while CCL5 was negatively correlated. (I) Comparison of albumin, CRP, fibrinogen, and transthyretin levels in patients with surgery durations above and below the median. (J-L) Inflammatory markers in blood, tumor, and ascitic fluid were stratified by surgery duration, divided into above or below the median of 336 minutes. Statistical significance was determined using Pearson correlation coefficient, with p-values indicated in the panels. Data represent 40 independent samples.

The concentration of 10 inflammatory cytokines and 2 adhesion molecules were quantified in tumor tissue, ascites fluid and blood biopsies obtained from patients with advanced ovarian cancer in the beginning of the cytoreductive surgery. The relationships between soluble factors levels and surgical complexity were evaluated. we analyzed the correlation between soluble factors and surgery duration for a general evaluation of their clinical potential. In tumor samples (Fig. 1A-C), CXCL-12, VCAM-1, and ICAM-1 levels negatively correlated with surgery duration. Notably, IL-32 in ascites (Fig. 1D), positively correlated with longer surgery durations, however, IL32 expression was heterogenous, with some patients exhibited IL-32 while others did not. However, the expression of IL-32 was more common in the patients with longer surgery duration (not shown). Therefore, IL-32 can play a role in driving the local innate inflammation within the ascites contributing to increased surgical complexity. In blood (Fig. 1E-H), levels of CCL-2 and CXCL-12 positively correlated with longer surgery duration. Conversely, serum CCL-5 and VCAM-1 levels negatively correlated with surgery duration.

Subsequently, we evaluated several preoperative inflammatory markers utilized in clinical practice, including Albumin, CRP, Fibrinogen, and Transthyretin, for potential correlations with surgery duration. No significant correlation was found between these markers and the surgery duration (Sup. Fig. 1A-D). Taken together, our data indicate that elevated levels of inflammatory markers, particularly IL-32, VCAM-1, and ICAM-1, are associated with longer surgery durations. These findings suggest that both systemic and local inflammation play significant roles in ovarian cancer progression.

### VCAM-1 and ICAM-1 are predictive marker candidates of duration of surgery in advanced ovarian cancer

To further evaluate the potential of cytokines to predict the surgery duration, patients were stratified into two groups based on a surgery duration threshold of 336 minutes. Firstly, the clinical preoperative inflammatory markers were analyzed and there were no significant differences in the patient groups (Fig. 1I). These findings suggest that while these markers may be useful for other clinical purposes, they do not appear to predict the duration of the surgical procedure.

Since the preoperative markers were not useful, we focused on investigating our 12 candidates to see whether they can be utilized for predicting the surgery duration. In tumor (Fig. 1J) we found elevated ICAM-1 level in extended surgery durations, however this borderline association did not reach statistical significance. In ascites (Fig. 1K), low VCAM-1 and ICAM1- levels showed an association with surgeries exceeding 336 minutes whereas only VCAM1 has significant association. In blood (Fig. 1L), interestingly we found slightly different trend than in ascites but similar in tumor. The levels of VCAM-1 and ICAM-1 were significantly elevated in extended surgery durations. These findings indicate that elevated systemic inflammation, reflected by increased levels of adhesion molecules that regulate immune cell trafficking such as VCAM-1 and ICAM-1, is associated with greater surgical complexity and longer operative durations. This underscores their potential as valuable biomarkers for predicting surgical duration in patients with advanced ovarian cancer.

### Cytokine and soluble factor profiles as predictors of surgical complexity in tumor, ascites, and blood samples

**Fig. 2:**
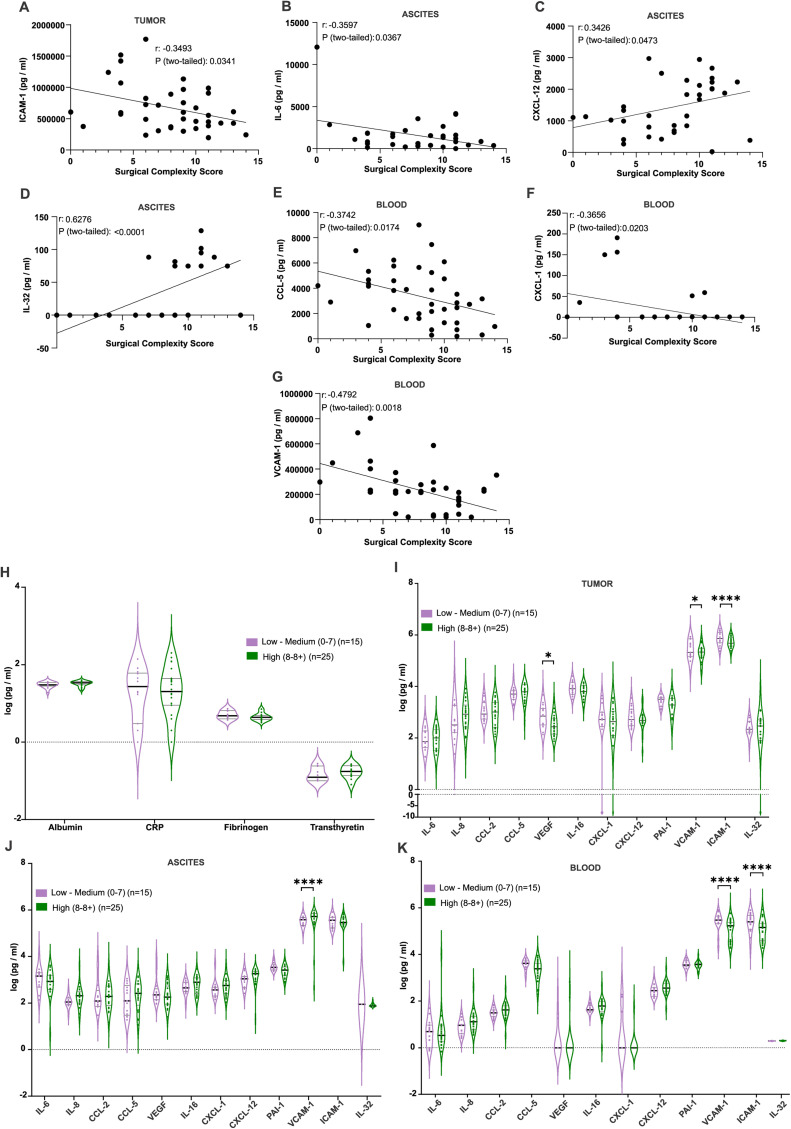
Higher surgery complexity scores are associated with differential levels of inflammatory markers in blood, tumor, and ascitic fluid. Significant differences were observed in the levels of VCAM-1, ICAM-1 in blood, VCAM1, ICAM1, and VEGF in tumour, only VCAM1 in ascites. (A) Tumor ICAM-1 and (B-C) ascitic IL-8 and CXCL12 levels showed correlations with the surgery complexity score. (D) IL-32 in ascitic fluid exhibited a strong positive correlation with the surgery complexity score (*r* = 0.6276, *p* < 0.0001). (E-G) In blood, negative correlations were found between surgery complexity score and CCL5, CXCL1, and VCAM-1 levels. (H Comparison of albumin, CRP, fibrinogen, and transthyretin levels between the two surgery complexity groups. (I-K) Inflammatory marker concentrations were measured in blood, tumor, and ascitic fluid, stratified by surgery complexity scores (low-medium: 0–7, *n* = 15; high: 8–8+, *n* = 25). Statistical significance was determined using Pearson correlation coefficient, with p-values indicated in the panels. Data represent 40 independent samples.

To understand the relation between these soluble factor and surgery complexity score, we further investigated the biopsies. In tumor samples (Fig. 2A), ICAM-1 levels presented a negative correlation with SCS. IL-6 level were negatively correlated with SCS (Fig. 2B). Conversely, in ascites CXCL-12 and IL-32 positively correlated with SCS. Furthermore, the patients with SCS lower than 6, did not have any detectable IL-32 expression in their ascites (Fig. 2C-D). In blood, several markers exhibited negative correlations with higher SCS (Fig. 2E-G). Specifically, CCL-5, CXCL-1, and VCAM-1 levels are inversely correlated with SCS.

We further examined the potential relationship between preoperative inflammatory markers and SCS, including Albumin, CRP, Fibrinogen, and Transthyretin (Fig. 2H). However, no significant correlation was found between these markers and the SCS (Sup. Fig. 2A-D), indicating that these markers are not reliable predictors of surgical complexity in the setting of advanced ovarian cancer.

### High VCAM-1 and ICAM-1 levels are associated with surgery complexity score

Subsequently, we assessed the relationship between the inflammatory cytokine and adhesion molecule levels in the tissue. In tumor (Fig. 2I), high levels of VEGF, VCAM-1 and ICAM-1 were significantly associated with low-medium SCS. A opposite pattern was observed in ascites samples (Fig. 2B), where low VCAM-1 level was significantly associated with low-medium SCS. However, in blood (Fig. 2C), we observed the same trend again as in tumor where high VCAM-1 and ICAM-1 levels showed a significant association with a low-medium SCS.

These findings also suggest that specific inflammatory markers, particularly VCAM-1, ICAM-1 may serve as indicators of surgery complexity in ovarian cancer patients. Elevated levels of these markers in blood, tumor, and ascites were associated with either a less complex or more complex surgical approach, depending on the marker and tissue type. These findings highlight the role of inflammation in influencing the complexity of cytoreductive surgery. These markers provide insight into the inflammatory processes influencing cytoreductive surgery outcomes and could assist in refining preoperative planning to optimize patient care in advanced ovarian cancer.

In the multivariable regression model, we tested the association between these two cytokines (VCAM-1 and ICAM-1) and surgical extent including the only clinical variables that may be known prior to the surgical procedure and is related to surgical extent. In the univariate analysis a higher level of VCAM-1 in blood was associated reduced odds of extensive surgery, Odds Ratio (OR) 0.16 (95 % CI, 0.03-0.96; *p*=0.045). However, this association did not remain after adjustment, OR 0.18 (95 % CI, 0.03-1.06; p=0.058).

The VCAM-1, ICAM-1 levels and FIGO stage by surgical extent are presented in Supplementary Table 1.

### Elevated VCAM-1 and ICAM-1 levels are associated with ovarian cancer recurrence

To explore the role of cytokines in advanced ovarian cancer recurrence, we analyzed the expression of 12 soluble factors in tumor tissue, ascitic fluid, and blood from patients with and without recurrence, aiming to uncover potential biomarkers and mechanisms underlying tumor progression.

**Fig. 3:**
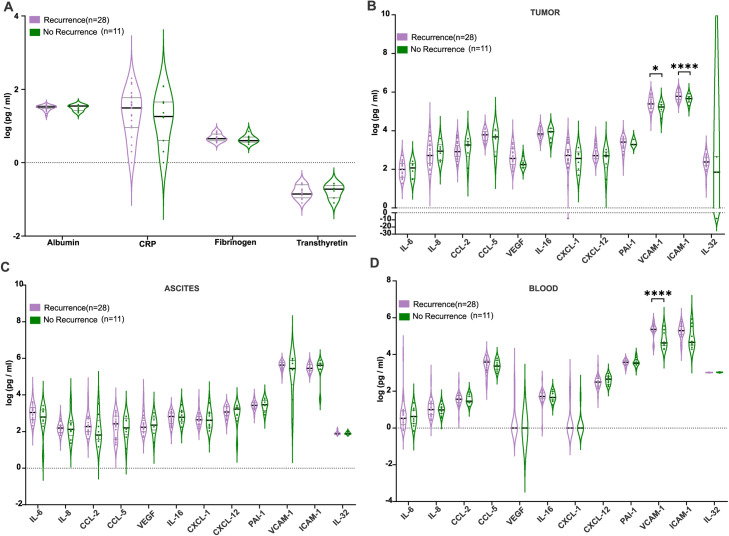
Patients with recurrence exhibit distinct inflammatory marker profiles compared to those without recurrence. (A) Comparison of albumin, CRP, fibrinogen, and transthyretin levels in patients with surgery durations above and below the median. No significant associations were observed between these markers and recurrence (B) In tumor samples, VCAM-1 and ICAM-1 levels were elevated in the recurrence group (*p < 0.05, ****p < 0.0001). (C) Ascitic fluid showed no statistically significant differences between recurrence and no-recurrence groups, although trends in inflammatory marker levels were observed (D) In blood, levels of ICAM-1 were significantly higher in patients with recurrence (*n* = 28) compared to those without recurrence (*n* = 11) (****p < 0.0001). Statistical comparisons were made using ANOVA test, and data represent 40 independent samples.

Firstly, we evaluated several preoperative inflammatory markers utilized in clinical practice, including Albumin, CRP, Fibrinogen, and Transthyretin, for potential correlations with recurrence (Fig. 3A). However, no significant associations were found between these markers and recurrence, although they may have utility in other clinical contexts, they are not effective predictors of disease relapse.

Our comparative analysis revealed significant variations in the expression of inflammatory and adhesion molecules. In tumor tissues, both VCAM-1 and ICAM-1 levels were significantly elevated in the recurrence group (Fig. 3B). In ascitic fluid, although VCAM-1 levels were higher in recurrent cases, the difference was not statistically significant (Fig. 3C). In blood, VCAM-1 levels were markedly higher in patients with recurrence compared to those without recurrence, while ICAM-1 levels showed no statistically significant differences (Fig. 3A). We therefore tested the association between these two cytokines (VCAM-1 and ICAM-1) and recurrence in a regression model including only clinical variables that may be known prior to the surgical procedure and related to oncologic outcome. After adjustment we observed that a higher level of VCAM-1 in blood prior to surgery was significantly associated with recurrence, Odds Ratio (OR) 10.1 (95 % CI, 1.30 to 77.8: *p*=0.027).

The VCAM-1, ICAM-1 levels and clinical characteristics by recurrence are presented in Supplementary Table 2.

Our findings suggest that VCAM-1 and ICAM-1 play a role in ovarian cancer recurrence, with VCAM-1 significantly elevated in tumor tissue and blood of recurrent cases. While ICAM-1 was higher in tumor tissue, its systemic relevance remains unclear. Preoperative inflammatory markers showed no predictive value for recurrence. These findings suggest that VCAM-1 could serve as an indicator of relapse and a promising therapeutic target, warranting further investigation into its role in ovarian cancer progression and recurrence.

### Receiver Operating Characteristic (ROC) analysis showed blood VCAM-1 levels excellently predict clinical outcome

**Fig. 4:**
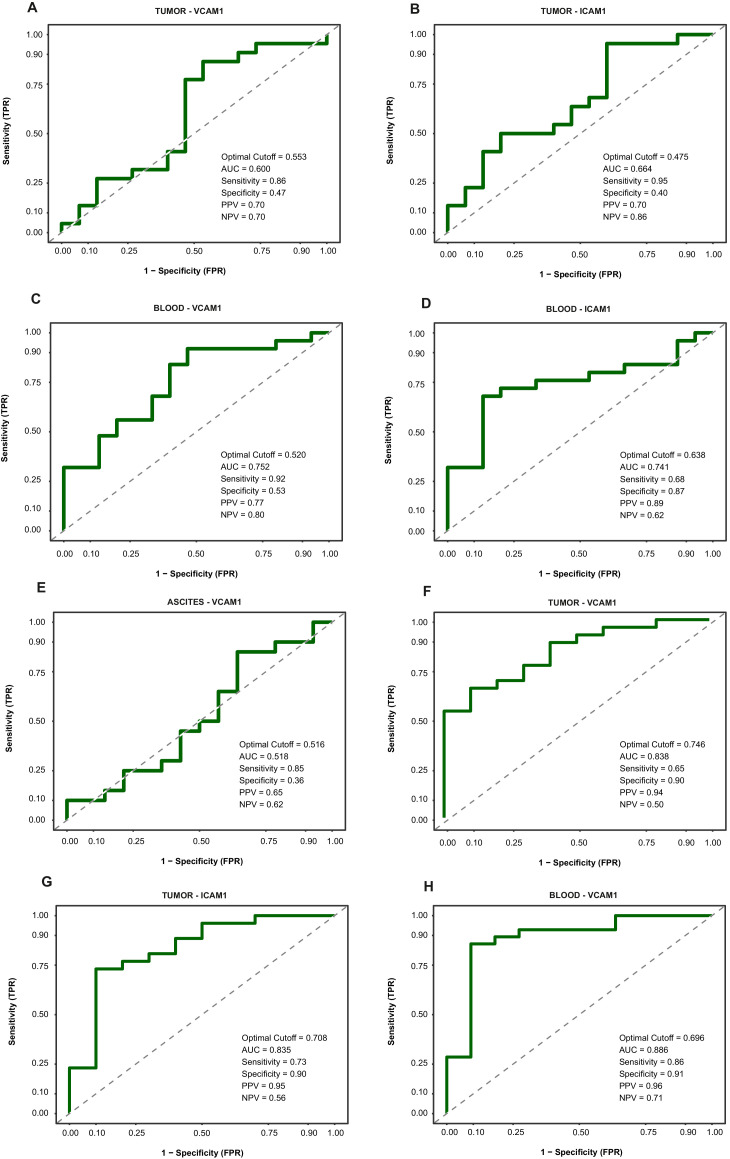
ROC curve analyses of VCAM-1 and ICAM-1 to predict surgical complexity and recurrence, adjusted for clinical variables. **A-E.** ROC curves evaluating the performance of VCAM-1 and ICAM-1 levels in tumor, blood and ascites samples for predicting high surgical complexity scores adjusted for preoperative suspected FIGO stage (III *vs.* IV). (A) Tumor VCAM-1, (B) Tumor ICAM-1, (C) Blood VCAM-1, (D) Blood ICAM-1, (E) Ascites VCAM-1. **F–H.** ROC curves assessing VCAM-1 and ICAM-1 as biomarkers for disease recurrence adjusted for clinical variables that may be known prior to the surgical procedure: Age (years, continuous), ECOG performance status (0, 1 or 2), BRCA mutation in tumour (yes *vs.* no) Preoperative suspected FIGO stage (III *vs.* IV) . (F) Tumor VCAM-1, (G) Tumor ICAM-1, (H) Blood VCAM-1. All values represent adjusted models. The AUC, optimal cut-off, sensitivity, and specificity values are indicated on each plot. *Abbreviations:* ROC, Receiver operating characteristic; VCAM-1, Vascular Cell Adhesion Molecule 1; ICAM-1, Intercellular Cell Adhesion Molecule 1; AUC, Area Under Curve; PPV, Positive Predictive Value; NPV, Negative Predictive value; TPR; True Positive Rate; FPR, False Positive Rate; FIGO, International Federation of Gynecology and Obstetrics; ECOG, Eastern Cooperative Oncology Group performance status; BRCA, BReast CAncer gene 1 or 2.

Adjusted ROC curves for the outcome *surgical complexity* are shown in Fig. 4. In tumor tissue, VCAM-1 and ICAM-1 demonstrated acceptable predictive performance, Area Under the Curve (AUC)=0.600 with cutoff point of 0.553 and AUC=0.664 with cutoff point of 0.475 (Fig. 4A and B). Improved performance was observed in blood VCAM-1 and ICAM-1, AUC=0.752 with cutoff point of 0.520, and AUC=0.741 with cutoff point 0.638 respectively (Fig. 4C and 4D).

In contrast, no predictive performance was seen in ascites, AUC=0.518 with cutoff point of 0.516 (Fig. 4E).

For the outcome *tumor recurrence*, both VCAM-1 and ICAM-1 showed good performance in tumor tissue, AUC= 0.838 with cutoff point of 0.746 and AUC=0.835 with cutoff point of 0.708 respectively (Fig. 4F and G). VCAM-1 in blood demonstrated exceptional predictive capability, AUC=0.886 with cutoff point of 0.696 (Fig. 4H). These results suggest the potential of VCAM-1 in blood as a preoperative predictive biomarker for clinical outcomes.

All above unadjusted results are provided in supplementary Figure 3.

## Discussion

This study provides valuable insights into the role of inflammatory markers in predicting surgical complexity and recurrence in advanced-stage ovarian cancer. We identified VCAM-1, ICAM-1, CXCL-12, and IL-32 as key markers correlating with surgical duration, complexity, and recurrence. Elevated levels of VCAM-1 and ICAM-1, particularly in tumor tissue, were strongly associated with recurrence, underscoring their potential as biomarkers and therapeutic targets. Notably, higher blood concentrations of VCAM-1 and ICAM-1 correlated with lower to medium surgical complexity scores (SCS), while elevated VCAM-1 levels in ascitic fluid were linked to longer surgical durations and high SCS. These findings highlight the dual role of these markers, both systemically and within the tumor microenvironment, in predicting surgical outcomes and contributing to tumor progression.

VCAM-1 and ICAM-1 mediate tumor cell adhesion to endothelial cells and the extracellular matrix, playing key roles in tumor progression, metastasis, and inflammation. In our study, preoperative blood and tumor levels of VCAM-1 and ICAM-1 were lower in high SCS patients compared to low-medium SCS cases, while VCAM-1 levels in ascitic fluid were elevated in high SCS patients. This inverse relationship may reflect compartmentalized inflammation, with increased local shedding of VCAM-1 into ascites due to a more inflamed tumor microenvironment. Lower systemic levels could result from sequestration within the ascites or reduced systemic inflammation due to immune exhaustion or tumor-mediated immunosuppression. These findings suggest that localized inflammation and adhesion molecule dynamics in the peritoneal cavity drive surgical complexity, while systemic levels may not fully reflect local disease burden. Elevated VCAM-1 in ascites highlights its role in tumor-endothelial interactions and metastatic spread, contributing to surgical challenges. This compartment-specific behavior underscores the importance of evaluating both systemic and local biomarkers to predict surgical outcomes and disease progression.

By identifying patients who are more likely to require complex surgical procedures or have a poor prognosis, the assessment of VCAM-1, ICAM-1, from easily collected liquid or tumour biopsies before the surgical procedure would contribute as important additional variables to be considered in the overall assessment of treatment recommendation. In the multivariable regression including clinical variables we observed VCAM-1 in blood as a particularly interesting biomarker that may be associated with clinical outcomes. However, these results should be interpreted with considerable caution due to the limited sample size and low event rate. Furthermore, even after adjustment, blood VCAM-1 demonstrated excellent predictive performance for recurrence, with an AUC exceeding 0.85, supporting its potential as a candidate for further prospective validation in adequately powered clinical studies. Therapeutically, targeting the inflammatory pathways associated with VCAM-1 and ICAM-1 offers a promising approach to prevent recurrence and improve patient outcomes [[30], [31], [32], [33], [34]]. Interventions aimed at inhibiting the expression or function of these adhesion molecules may disrupt tumor cell adhesion, invasion, and immune evasion, thereby reducing recurrence and improving prognosis [[31], [32], [33]]. Our findings underscore the potential utility of these markers not only as prognostic indicators but also as therapeutic targets. Previous studies have suggested the therapeutic relevance of VCAM-1 and ICAM-1 in various solid malignancies. For example, studies in breast, lung and colorectal cancers have demonstrated similar associations between these markers and metastatic behavior [31,35,36]. However, the present study extends these findings to ovarian cancer by integrating data from multiple sample types—blood, tumor, and ascites—and correlating these markers with both surgical complexity and recurrence. This multifaceted approach allows for a more comprehensive understanding of the role of inflammatory markers in ovarian cancer.

Despite our findings, several limitations should be acknowledged. First, the study was constrained by a small sample size and lack of longitudinal follow-up, limiting our ability to evaluate the long-term prognostic significance of the identified biomarkers. Second, the regression analysis should be interpreted with caution due to the limited number of events, which impact the stability and precision of the model as exemplified by the confidence interval of the estimate. Additionally, no a priori power or sample size calculations were conducted, and given the exploratory nature of the study, post hoc power analysis was inadequate. Nevertheless, the prospective collection of samples and clinical data within the context of a phase III clinical trial adds rigor to the study.

## Conclusions

In conclusion, our study highlights the significant role of inflammatory markers and underscores their potential as innovative clinical tools for patient stratification to surgical treatment in advanced ovarian cancer. The adhesion molecules VCAM-1 and ICAM-1 emerged as the strongest candidates, outperforming cytokines and other clinical markers in predicting surgical complexity and recurrence. Notably, liquid biopsies, which are less invasive and easy to perform, demonstrated superior performance over traditional tumor tissue biopsies in identifying these markers. We identified promising biomarkers that warrant further investigation to uncover the mechanistic pathways through which these inflammatory markers influence tumor progression. Moreover, validation in larger adequately powered prospective studies is required to determine their accuracy in predicting clinical outcomes, to improve patient selection to treatment.

## Ethics approval and consent to participate

This study was approved by the Swedish Ethical Review Authority (Dnr: 2019-05149). All participating patients provided an informed written consent.

## Consent for publication

Not applicable.

## Availability of data and materials

The datasets used and/or analysed during the current study are available from the corresponding author on reasonable request.

## CRediT authorship contribution statement

**Okan Gultekin:** Methodology, Formal analysis, Visualization, Conceptualization, Software, Investigation, Data curation, Writing – original draft. **Jordi Gonzalez-Molina:** Visualization, Investigation, Supervision, Methodology, Writing – review & editing. **Dhifaf Sarhan:** Visualization, Formal analysis, Writing – review & editing, Supervision. **Nina Groes-Kofoed:** Resources, Investigation, Data curation, Project administration, Formal analysis. **Mahmood Ul Hassan:** Methodology, Software, Validation, Writing – review & editing, Visualization. **Kaisa Lehti:** Writing – review & editing, Resources, Investigation, Conceptualization, Supervision, Project administration, Funding acquisition. **Sahar Salehi:** Writing – original draft, Supervision, Data curation, Writing – review & editing, Project administration, Conceptualization, Resources, Investigation, Visualization, Funding acquisition.

## Declaration of competing interest

The authors declare that they have no known competing financial interests or personal relationships that could have appeared to influence the work reported in this paper.

## Appendix Supplementary materials

Supplementary Figure 1: Exploration of inflammatory markers relative to surgery duration. (A–D) Correlations of preoperative albumin (A), CRP (B), fibrinogen (C), and transthyretin () levels with surgery duration. No significant associations were observed between these markers and surgery duration. Data were represented with results as means ± SEM or scatterplots with correlation coefficients (r) and p-values. Image, application 1

Supplementary Figure 2: Analysis of inflammatory markers stratified by surgery complexity scores. (H) Comparison of albumin, CRP, fibrinogen, and transthyretin levels between the two surgery complexity groups. (I–L) Correlations of preoperative albumin (I), CRP (J), fibrinogen (K), and transthyretin (L) levels with surgery complexity scores. Data were represented as means ± SEM or scatterplots displaying correlation coefficients (r) and p-values. No significant trends were observed between nutritional markers and surgery complexity scores. Image, application 2

Supplementary Figure 3. ROC curve analyses of VCAM-1 and ICAM-1 to predict surgical complexity and recurrence, unadjusted **A-E.** ROC curves evaluating the performance of VCAM-1 and ICAM-1 levels in tumor, blood, and ascites samples for predicting high surgical complexity scores. (A) Tumor VCAM-1, (B) Tumor ICAM-1, (C) Blood VCAM-1, (D) Blood ICAM-1, (E) Ascites VCAM-1. **F–H.** ROC curves assessing VCAM-1 and ICAM-1 as biomarkers for disease recurrence. (F) Tumor VCAM-1, (G) Tumor ICAM-1, (H) Blood VCAM-1. All values represent adjusted models. The AUC, optimal cut-off, sensitivity, and specificity values are indicated on each plot. *Abbreviations:* ROC, Receiver operating characteristic; VCAM-1, Vascular Cell Adhesion Molecule 1; ICAM-1, Intercellular Cell Adhesion Molecule 1; AUC, Area Under Curve; PPV, Positive Predictive Value; NPV, Negative Predictive value; TPR; True Positive Rate; FPR, False Positive Rate. Image, application 3

Image, application 4

Image, application 5
