# Supplementary figures and images for: Systemic and tumor-specific inflammatory markers VCAM-1 and ICAM-1 as indicators of extent of surgery and oncologic outcome in advanced ovarian cancer
Source: Transl Oncol. 2025 Jul 12;59:102462. doi: 10.1016/j.tranon.2025.102462 (PMC12302516; doi:10.1016/j.tranon.2025.102462)

Supplementary Figure 1

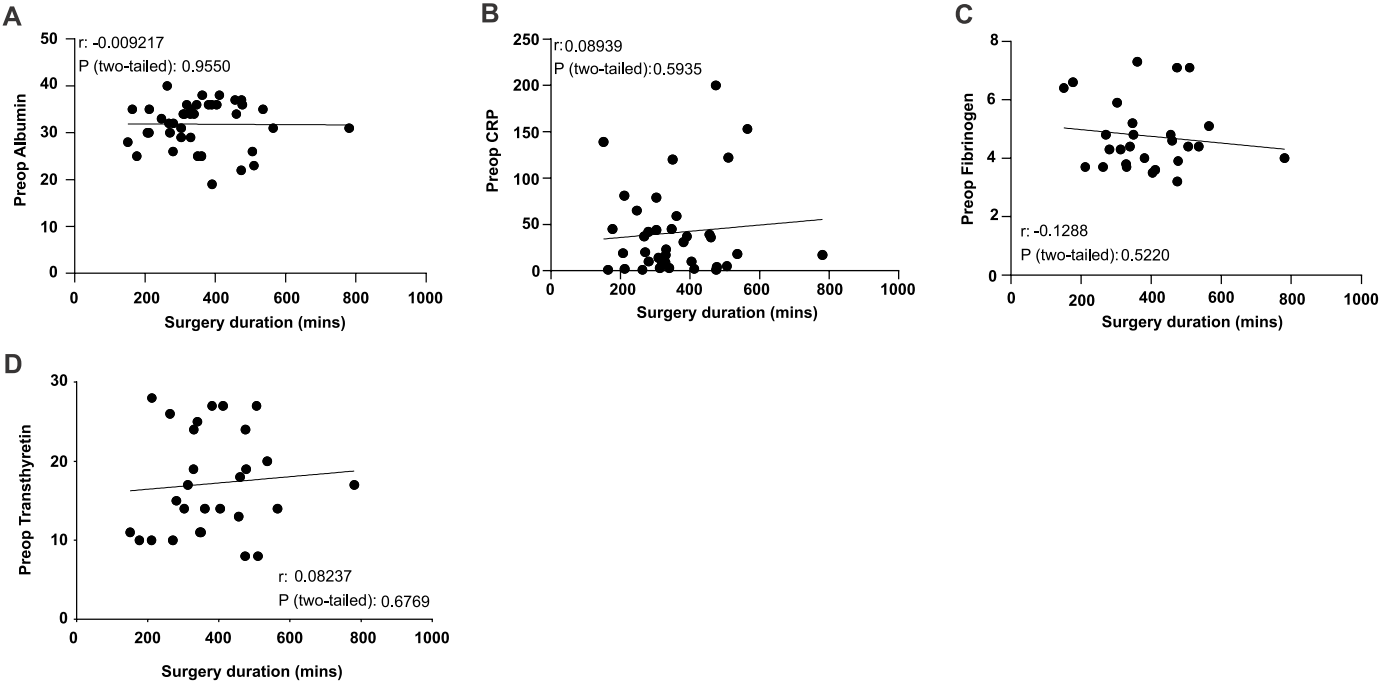

Supplement: Supplementary file 1 — Supplementary Figure 1: Exploration of inflammatory markers relative to surgery duration. (A–D) Correlations of preoperative albumin (A), CRP (B), fibrinogen (C), and transthyretin () levels with surgery duration. No significant associations were observed between these markers and surgery duration. Data were represented with results as means ± SEM or scatterplots with correlation coefficients (r) and p-values. [file mmc1.pdf]

Supplementary Figure 2

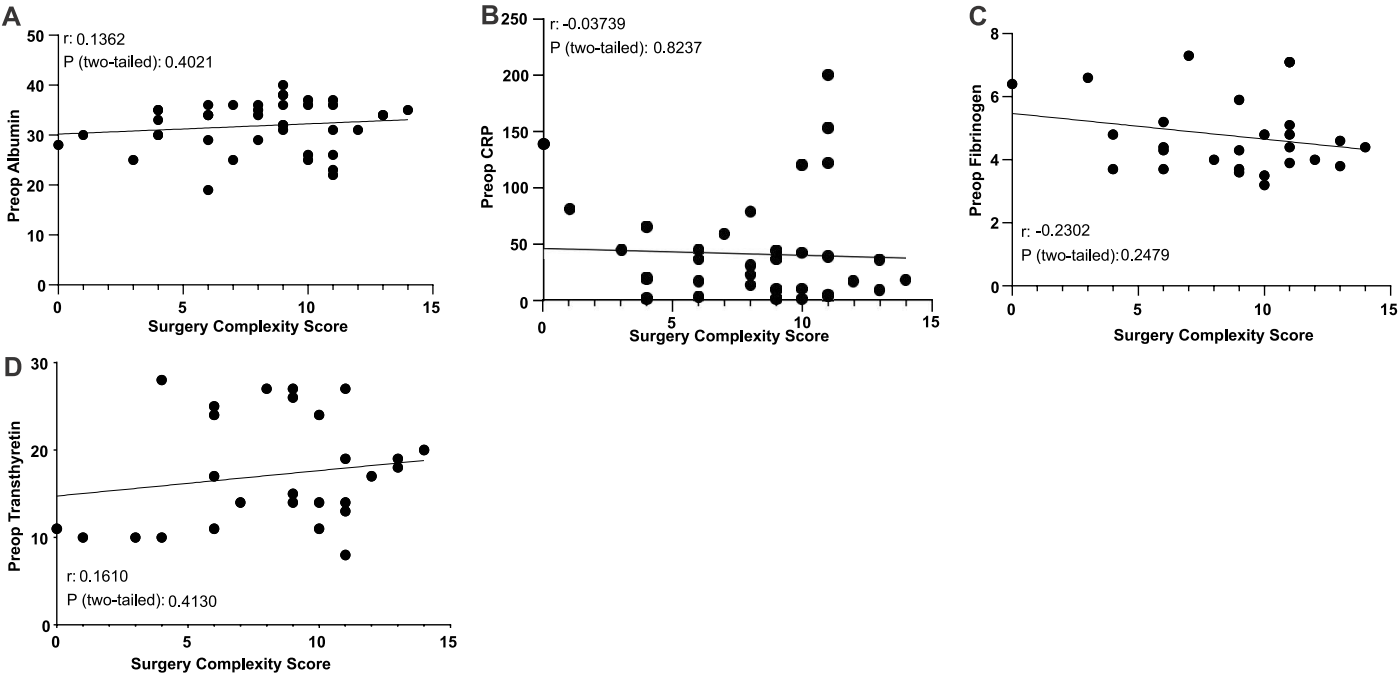

Supplement: Supplementary file 2 — Supplementary Figure 2: Analysis of inflammatory markers stratified by surgery complexity scores. (H) Comparison of albumin, CRP, fibrinogen, and transthyretin levels between the two surgery complexity groups. (I–L) Correlations of preoperative albumin (I), CRP (J), fibrinogen (K), and transthyretin (L) levels with surgery complexity scores. Data were represented as means ± SEM or scatterplots displaying correlation coefficients (r) and p-values. No significant trends were observed between nutritional markers and surgery complexity scores. [file mmc2.pdf]

Supplementary Figure 3

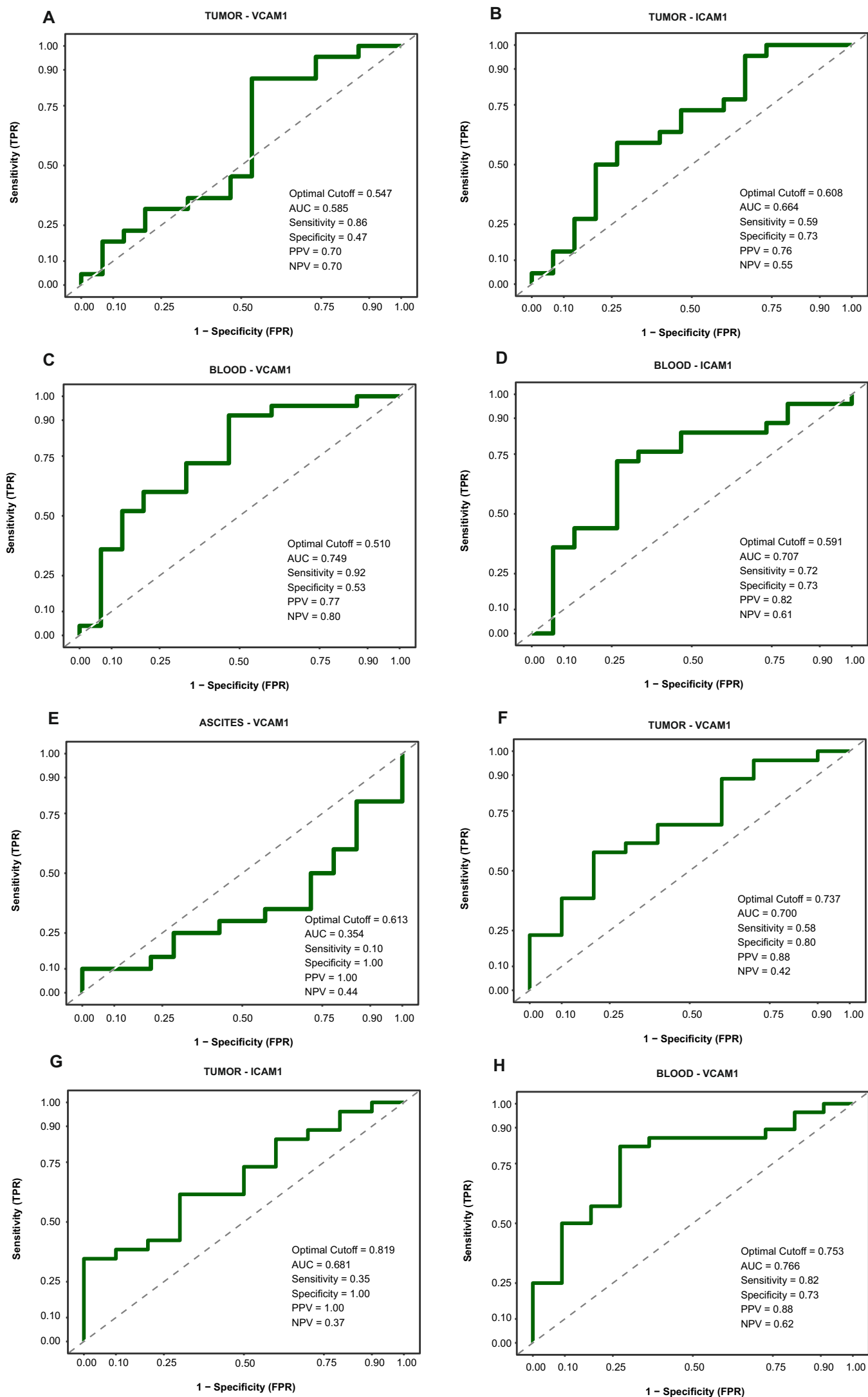

Supplement: Supplementary file 3 — Supplementary Figure 3. ROC curve analyses of VCAM-1 and ICAM-1 to predict surgical complexity and recurrence, unadjusted A-E. ROC curves evaluating the performance of VCAM-1 and ICAM-1 levels in tumor, blood, and ascites samples for predicting high surgical complexity scores. (A) Tumor VCAM-1, (B) Tumor ICAM-1, (C) Blood VCAM-1, (D) Blood ICAM-1, (E) Ascites VCAM-1. F–H. ROC curves assessing VCAM-1 and ICAM-1 as biomarkers for disease recurrence. (F) Tumor VCAM-1, (G) Tumor ICAM-1, (H) Blood VCAM-1. All values represent adjusted models. The AUC, optimal cut-off, sensitivity, and specificity values are indicated on each plot. Abbreviations: ROC, Receiver operating characteristic; VCAM-1, Vascular Cell Adhesion Molecule 1; ICAM-1, Intercellular Cell Adhesion Molecule 1; AUC, Area Under Curve; PPV, Positive Predictive Value; NPV, Negative Predictive value; TPR; True Positive Rate; FPR, False Positive Rate. [file mmc3.pdf]
