# Supplementary material for: Systemic and tumor-specific inflammatory markers VCAM-1 and ICAM-1 as indicators of extent of surgery and oncologic outcome in advanced ovarian cancer
Source: Transl Oncol. 2025 Jul 12;59:102462. doi: 10.1016/j.tranon.2025.102462 (PMC12302516; doi:10.1016/j.tranon.2025.102462)
Supplement: Supplementary file 4 [file mmc4.docx]

| **Supplementary Table 1. VCAM-1, ICAM-1 levels^*^ and FIGO stage of included patients with advanced ovarian cancer undergoing cytoreductive surgery with curative intent, by surgical extent.** | | | |
| --- | --- | --- | --- |
| **Variable** | **High SCS**^1^  ***n* = 15** | **Low/medium SCS**  ***n* = 25** | **p-value***^2^* |
| VCAM in tumour, median (IQR^*^) | 5.32 (5.17, 5.84) | 5.34 (5.14, 5.48) | 0.400 |
| ICAM in tumour, median (IQR) | 5.85 (5.69, 6.09) | 5.68 (5.57, 5.88) | 0.098 |
| VCAM in blood, median (IQR) | 5.47 (5.34, 5.65) | 5.24 (4.58, 5.37) | 0.008 |
| ICAM in blood, median (IQR) | 5.40 (5.07, 5.66) | 5.16 (4.63, 5.30) | 0.030 |
| VCAM in ascites, median (IQR) | 5.58 (5.38, 5.71) | 5.71 (5.45, 5.80) | 0.158 |
| Preop FIGO stage, no. (%)      III      IV | 10 (67)  5 (33) | 21 (84)  4 (16) | 0.255 |
| ***Abbreviations:*** VCAM, Vascular Cell Adhesion Molecule; ICAM, Intercellular Adhesion Molecule; FIGO, International Federation of Gynecology and Obstetrics; SCS, Surgical Complexity Score; IQR, Inter Quartile Range.  *** pg/mL  ^1^ Surgical Complexity Score according to Aletti et al.  *^2^*Wilcoxon rank sum exact test; Wilcoxon rank sum test; Fisher's exact test  ^*^2^nd^ to 3^rd^ quartile. | | | |
