# Supplementary material for: Systemic and tumor-specific inflammatory markers VCAM-1 and ICAM-1 as indicators of extent of surgery and oncologic outcome in advanced ovarian cancer
Source: Transl Oncol. 2025 Jul 12;59:102462. doi: 10.1016/j.tranon.2025.102462 (PMC12302516; doi:10.1016/j.tranon.2025.102462)
Supplement: Supplementary file 5 [file mmc5.docx]

| **Supplementary Table 2. VCAM-1, ICAM-1 levels^*^ and characteristics of included patients with advanced ovarian cancer undergoing cytoreductive surgery with curative intent, by recurrence^1^.** | | | |
| --- | --- | --- | --- |
| **Variable** | **No Recurrence**  ***n* = 11** | **Recurrence**  ***n* = 29** | **p-value***^2^* |
| VCAM in tumour, median (IQR^*^) | 5.23 (4.86, 5.33) | 5.38 (5.17, 5.71) | 0.052 |
| ICAM in tumour, median (IQR) | 5.66 (5.53, 5.83) | 5.78 (5.63, 5.99) | 0.078 |
| VCAM in blood, median (IQR) | 4.63 (4.50, 5.35) | 5.37 (5.32, 5.49) | 0.008 |
| ICAM in blood, median (IQR) | 4.66 (4.51, 5.54) | 5.29 (5.14, 5.50) | 0.074 |
| VCAM in ascites, median (IQR) | 5.45 (5.37, 5.87) | 5.62 (5.50, 5.77) | 0.730 |
| Age (years), median (IQR) | 60 (52, 67) | 71 (62, 74) | 0.036 |
| ECOG performance status, no. (%)      0      1      2 | 7 (64)  4 (36)  0 (0) | 15 (52)  11 (38)  3 (10) | 0.765 |
| BRCA mutation in tumor, no. (%)      No      Yes | 6 (55)  5 (45) | 23 (82%)  5 (18%) | 0.109 |
| Preoperative FIGO stage, no. (%)      III      IV | 11 (100)  0 (0) | 20 (69)  9 (31) | 0.043 |
| ***Abbreviations:*** VCAM, Vascular Cell Adhesion Molecule; ICAM, Intercellular Adhesion Molecule; IQR, Inter Quartile Range; ECOG, Eastern Cooperative Oncology Group performance status; BRCA, BReast CAncer gene 1 or 2; FIGO, International Federation of Gynecology and Obstetrics.  *** pg/mL  ^1^ After a median follow-up time of 34 months (IQR 29-43)  *^2^*Wilcoxon rank sum exact test; Wilcoxon rank sum test; Fisher's exact test  ^*^2^nd^ to 3^rd^ quartile. | | | |
